# Innovative integrative bedside teaching model improves tutors’ self-assessments of teaching skills and attitudes

**DOI:** 10.3402/meo.v21.30526

**Published:** 2016-02-16

**Authors:** Itai Gat, Liat Pessach-Gelblum, Gili Givati, Nadav Haim, Shani Paluch-Shimon, Avraham Unterman, Yochay Bar-Shavit, Galit Grabler, Doron Sagi, Anat Achiron, Amitai Ziv

**Affiliations:** 1 MSR-Israel Center for Medical Simulation, Sheba Medical Center, Tel Hashomer, Israel; 2 Pinchas Borenstein Talpiot Medical Leadership Program, Sheba Medical Center, Tel Hashomer, Israel; 3 Sackler Faculty of Medicine, Tel Aviv University, Tel Aviv, Israel; 4 Department of Neurology, Sheba Medical Center, Tel Hashomer, Israel; 5 Department of General Surgery, Sheba Medical Center, Tel Hashomer, Israel; 6 Division of Oncology, Sheba Medical Centre, Tel Hashomer, Israel; 7 Multiple Sclerosis Center, Sheba Medical Center, Tel Hashomer, Israel

**Keywords:** medical education, standardized patients, clinical teaching, physician–patient communication, role model

## Abstract

**Introduction:**

Patient bedside is the ideal setting for teaching physical examination, medical interviewing, and interpersonal skills. Herein we describe a novel model for bedside teaching (BST) practiced during tutor training workshop and its resulting effect on practitioners’ self assessment of teaching skills and perceptions.

**Methods:**

One-day tutor training workshop included theoretical knowledge supplementation regarding tutors’ roles as well as implementing practical tools for clinical education, mainly BST model. The model, which emphasizes simultaneous clinical and communication teaching in a stepwise approach, was practiced by consecutive simulations with a gradual escalation of difficulty and adjusted instruction approaches. Pre- and post-workshop-adjusted questionnaires using a Likert scale of 1 to 4 were completed by participants and compared.

**Results:**

Analysis was based on 25 out of 48 participants who completed both questionnaires. Significantly improved teaching skills were demonstrated upon workshop completion (mean 3.3, SD 0.5) compared with pre-training (mean 2.6, SD 0.6; *p<*0.001) with significant increase in most examined parameters. Significantly improved tutor's roles internalization was demonstrated after training completion (mean 3.7, SD 0.3) compared with pre-workshop (mean 3.5 SD 0.5; *p=*0.002).

**Discussion:**

Successful BST involves combination of clinical and communication skills. BST model practiced during the workshop may contribute to improved teaching skills in this challenging environment.

It seems obvious that since clinical practice involves the diagnosis and treatment of patients with problems, teaching of clinical medicine should be carried out on real patients with real problems (1). Patients’ bedside is the ideal setting for teaching physical examination, medical interviewing, and interpersonal skills. While demonstrating physical findings is the most commonly reported objective for bedside visits, difficult parts of the interview (such as inquiring about depression), handling emotions (anger, fear, etc.), as well as counseling and supporting the patient can be demonstrated as necessary for professionalism and humanism (2). Nair et al. emphasized the importance of bedside teaching (BST), as 100% of learners (students, interns, and residents) reported that BST is an effective way to learn professional skills (3).

In spite of the crucial importance of BST, it seems that BST does not necessarily fulfill its entire potential during clinical education. In previous cross-sectional study, only 48% of learners reported they received ‘enough’ BST (3). Many obstacles in the clinical environment make BST challenging, such as time constraints, patient-related challenges (short hospital stay, unwillingness to participate in patient encounter), lack of clear objectives and expectations, lack of active learners’ participation, as well as an uncomfortable physical environment for clinical teaching (4, 5). In order to overcome these obstacles, the clinical teacher should adopt effective tools such as control of session, clarification of goals, and promoting positive learning climate (4).

Given its potential benefit, physicians and medical teachers should develop improved approaches and methods to enhance BST (1). Various ideas have been adopted in order to improve BST such as workshops assigned to develop physicians teaching skills (6, 7), assessments for patient selection for BST (8), and models for BST performance suggested by Cox (9, 10) and Janicik and Fletcher (11). Both models emphasize the importance of preparation before and debriefing after BST in relation to communicating with the patient during BST.

A novel workshop for tutors has been performed in the Israel Center for Medical Simulation since 2010 (12). Our perception of the ‘ideal’ tutor is as a well-experienced clinician with high communication skills and great passion for medical education. Furthermore, we believe that clinical education (and BST in particular) should simultaneously include clinical and communication skills (13) while coordinating efficiently between students and patient (Fig. 1). Accordingly we developed a novel model for BST based on our tutors training experience. The model develops previous BST models a further step forward by the incorporation of teaching tools and communication skills into the different stages of BST. During the workshop, participants practiced and exercised the model with standardized patients (SP) in order to become familiar with the different BST stages and the suitable skills in each step. Herein we describe the model and its impact on practitioners’ self assessment of teaching skills and abilities.

**Fig. 1:**
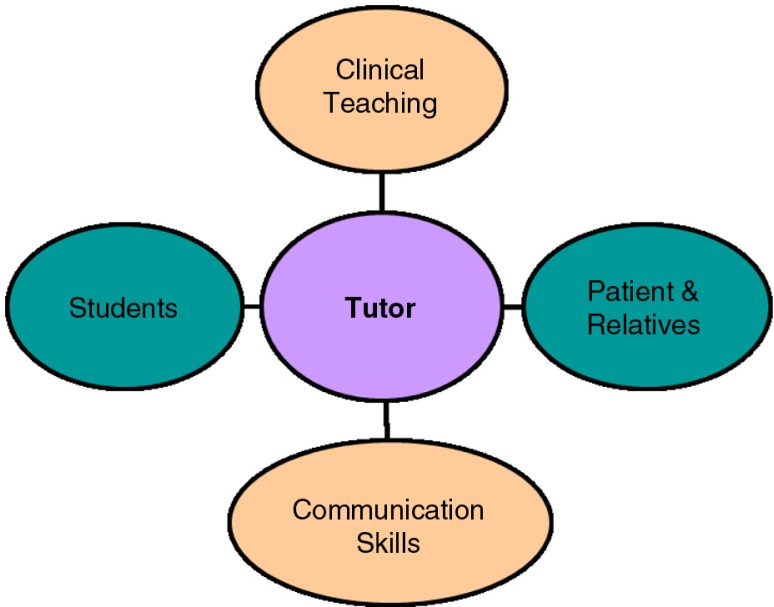
General perception of tutor's role during bedside teaching.

## Methods

### Population

Physicians from Sheba Medical Center, Israel, were referred to the workshop between November 2012 and April 2014 by their heads of departments with the intention of their acting as tutors for medical school students from Sackler Faculty of Medicine, Tel Aviv University, and St George's, University of London Medical Program at the University of Nicosia, Cyprus. We decided to focus training on main and long clerkships in which the tutor's impact on students’ education is high. Therefore, our participants were general surgeons, gynecologists, psychiatrists, pediatricians, and internal medicine physicians, without practitioners from sub-surgery departments and other short clerkships. Residents assignment as tutors (especially during advanced stages of residency) was relatively common as part of residents training and exposure to medical education, under the supervision of senior physician. All practitioners signed informed consent at the beginning of the workshop according to approved institutional review board (IRB).

### Workshop program and BST model

The 1-day tutor training workshop lasted 7 h and had two main objectives. Since there is no other instruction or preparation for physicians who intend to be tutors, the first goal was to supply theoretical background regarding tutor's function during medical education. We state that tutor's role combines teaching of clinical content (including clinical skills and theoretical knowledge) as well as communication skills (such as patient–physician relationship and coping with ethical issues). The second and main objective was to teach and exercise practical tools for effective BST. The workshop's curriculum correlates these two objectives: after a 1.5-h talk focused on theoretical background, most of the day was dedicated to integrating medical teaching skills, mainly by the adoption of the novel BST model.

The fundamental principal of the BST model is strict adherence to the three stages of clinical teaching – preparation, BST, and debriefing. Each stage has its own emphases and objectives (10, 11). The discrepancy between the ‘pre-’ and ‘post-’ patient encounter enables to focus BST on meeting objectives in order to accomplish a comprehensive and professional medical educational process.

#### Preparation (Fig. 2)

1. *Teacher* – Preparation is a key element to conduct effective rounds (14). The initial step is teacher's self-preparation including defining encounter objectives, which should correlate clinical curriculum (10), students’ level, and patient selection. During the model, we emphasize the importance of integration between clinical skills and communication abilities. Therefore, meeting's goals may include both clinical (e.g., assessment of patient with cirrhosis who has ascites and organomegaly) and communicational (e.g., coping with patient's frustration due to lack of improvement during hospitalization) aspects.
2. *Patient* – The main consideration for patient selection by the medical teachers is educational, mainly correlating patient's illness and lesson's topic. Additional categories are bio-psycho-social (e.g., language and verbal skills) and structural ( e.g., ability to contact patient) factors (8). Discussion with the patient prior to BST performance is mandatory in order to obtain consent, emphasize the importance of medical teaching, and define the encounter's objectives and schedule.
3. *Students* – The teacher should brief the students regarding clinical and communication aspects. All participants need to know encounter's goals and time constraints as well as each student's part during the encounter. Clarification should be made regarding communication limits such as postponing sensitive discussions (14).

**Fig. 2:**
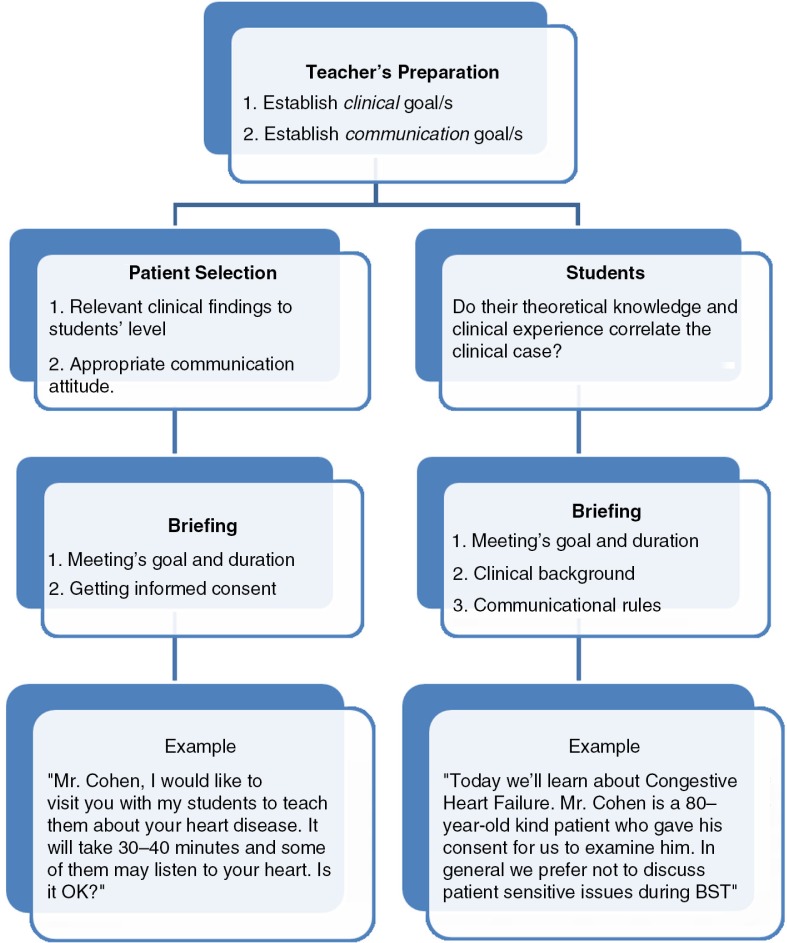
Preparation for BST.

Comprehensive preparation, which starts from the teacher then continues with patient and students briefing, is cost effective since it enables optimal clinical teaching and achievement of objectives during the encounter. Disruptive issues such as communication obstacles should be resolved prior to patient encounter. For example, it needs to be clarified that poor prognosis should be discussed in front of the patient only in rare and unique circumstances.

#### Bedside teaching

BST is performed after detailed preparation and needs to be focused on achieving determined goals. The instructor should coordinate simultaneously between the patient and students in aspects of both clinical teaching and interpersonal interaction. Therefore, any step during the encounter requires different teaching and communication skills as detailed in Table 1.

1. *
Introduction* – Although both patient and trainees are briefed prior to the meeting, the instructor should open the encounter with a short introduction regarding the objectives and time scale accompanied by introduction of all participants.
2. *Clinical teaching* – This part is the heart of medical teaching. During the session, a delicate balance between individual and team training should be maintained. Therefore, clinical teaching starts with training of a single student who was selected in advance and then shifts to group teaching. Interaction with the patient in that stage is empathetic but short in order to supply trainees fluent practice without disruptions.
  - *Individual instruction* – After introduction of the clinical student's assignment (‘we will practice respiratory system examination’), the previously elected student should perform the task. During student practice, the teacher may use leading questions (‘What is the next step after inspection?’), short demonstrations in order to emphasize specific elements, and gentle corrections as needed. Trainees’ experience during the clinical task is mandatory in order to develop professional skills and attitudes; therefore, teacher's interferences should be short and precise. Few short comments or questions targeted to other students may engage them and keep them focused, but the main emphasis should be on the practicing student.
  - *Team teaching* – After the student has accomplished his assignment, the personal teaching shifts to team training. Since performing the whole assignment by all trainees is impossible, the non-practicing students should focus only on the main essence of clinical lessons (‘Please listen to the decreased breathing sounds in Rt lower lobe’). Suitable communication tools in that stage are open questions leading to group discussion (‘Which other signs can be found in patients with Pneumothorax?’), encouraging quiet students, and control over dominant colleagues. Specifically, questions should correlate trainees’ level especially in heterogeneous groups composed of trainees in various training periods (students of different years, interns and/or residents). Less experienced participants should perform easier tasks at the beginning of group teaching followed by more difficult missions assigned by trainees at more advance stages. Likewise, the later may demonstrate clinical skills to the former as a method to engage all group members.
  The stage of clinical teaching may be repeated as necessary to meet goals. For example, BST with a patient suffering from ischemic heart disease may include history taking as well as physical examination of the cardiovascular system. In that case, history taking will be performed by one trainee leading to team discussion on that topic. Then, another student will perform a comprehensive physical examination, while his colleagues will examine only positive findings and discuss specific aspects of physical examination.
3. *Closure* – The teacher should summarize trainees’ assignments in relation to the encounter's goals as defined during meeting introduction. Then, the attention should turn to patient's point of view. The teacher should encourage the patient to share his personal experience during his hospitalization and illness. Contrary to the short interaction with the patient during clinical teaching, this stage is characterized by open questions directed to the patient and establishment of a less formal atmosphere. Teacher–patient interaction can be used as a role model for communication skills and humanistic attitude. Thereafter, time should be given to students’ questions and clarifications regarding the clinical task. Moreover, the teacher should encourage direct student–patient interactions in order to summarize the encounter from the clinical and communication aspects at the same time.

**Table 1:** Bedside teaching – step by step

Stage		Communication tools
Introduction		Acquaint students and patient, clarify meeting goals and schedule, ensure adequate physical conditions
Clinical teaching^a^	Individual instruction	Task presentation, leading questions, teacher's observation versus demonstration
	Group training	Open questions, team discussion, encourage quiet students, share positive findings on physical examination
Closure	Summarization	Task performance in relation to meeting goals
	Patient's input	Open question directed to patient, may focus on patient's personal life (emotions, experiences, and beliefs related to illness), less formal atmosphere
	Students’ questions	Open discussion, encourage direct patient–student interaction
	Thank patient!	

a Clinical teaching may be repeated as necessary according to meeting goals.

It should be noted that all contents during patient encounter should fulfill two conditions: First, patient persistence needs to be relevant and helpful. Second, the content needs to be relevant to session goals. However, bedside is the perfect venue for unrehearsed and unexpected triangular interactions between teacher, trainees, and patients. Physician teachers should capture such teachable moments and use them to reinforce messages regarding knowledge, skills, or attitudes (14).

#### Debriefing (Fig. 3)

BST should not be finished at the end of the patient encounter! Debriefing immediately after leaving the patient's room is mandatory in order to give feedback, perform complementary clinical discussion, and disclosure.

1. *Feedback* – It is an important medical teaching tool (15), which should ascertain whether encounter objectives were met. This session should remain brief and focus on the strengths and deficiencies. Similar to clinical teaching, feedback should start with the individual student who performed the clinical task. Self-analysis of positive and negative actions by the trainee is essential with applause for good work and correction of mistakes. Thereafter, team feedback is performed to improve the value of future patient encounters. Attention should be given to both clinical and communication aspects in order to enforce the combination of professionalism and humanism during interaction with patients.
2. *Clinical discussion related to discipline* – Clinical discussion outside patient room should integrate clinical information gathered from the patient with complementary examinations (laboratory, imaging, etc.) and theoretical knowledge. This is the appropriate setting to negotiate clinical aspects that do not require patient's presence (e.g., differential diagnosis) and to raise sensitive issues including prognosis.
3. *Disclosure* – Toward the end of the session, the teacher should implement an integrated summary of the whole meeting. Special emphasis should focus on the integration between theoretical data and clinical findings presented during the session in relation to session goals. Take home messages divided into clinical versus communication lessons should be reinforced, some of them specific to the meeting's topics and other general conceptions that may be implemented during different clinical circumstances.

**Fig. 3:**
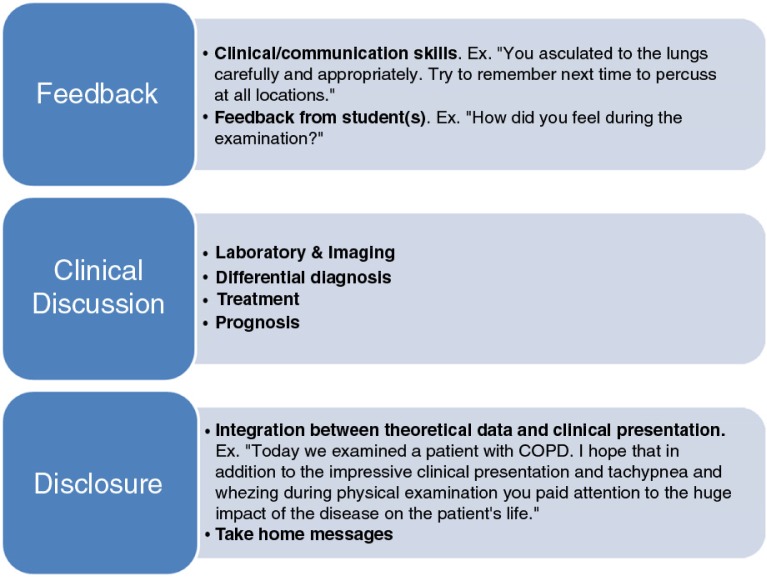
Debrief.

### Simulations

The BST model was practiced by three consecutive scenarios. In all simulations, one participant served as a tutor while others were ‘students’. ‘Students’ challenged the practicing tutor according to pre-scenario instructions such as performing disordered physical examination and avoiding hand washing.

Some general considerations directed us during scenario development. First, since BST is sophisticated and complex, we established a gradual escalation difficulty between scenarios. Accordingly, the first two scenarios included only the preparation and BST, while the third and last scenario included a comprehensive implementation of the model from briefing to debriefing. Specifically, the first simulation had simple single clinical objective (teaching basic neurological examination) with cooperative ‘patient’ in order to implement the BST model without communicational distractions. The second scenario had two clinical objectives (practice medical history taking as well as respiratory physical examination) accompanied with communicational challenge presented by the ‘patient’. The last simulation was the most provocative since the practitioner had to face combined clinical and communicational challenges and ethical dilemma presented by patient refuses to life-saving oncological treatment (Appendix 1). Second, workshop instructors attended scenario rooms and supervised participants’ performance similar to the way the tutor instructs students during real BST. We believe this kind of supervision supplies practitioners a role model, which is mandatory during fulfillment of tutor's role. Third, since the workshop addressed multi-disciplinary physicians and the focus of the workshop was on educational aspects, the clinical contents during scenarios were basic and suitable for all physicians.

### Tools and statistical analysis

Practitioners completed questionnaires specially designed for the workshop at the beginning and at the end of the day. The pre-questionnaire included 15 items on a 1 to 4 Likert scale. The reliability of the scale was 0.89. Two measures were calculated for each participant: (1) Instruction skills were based on eight items (1–8) and (2) Attitudes were based on seven items (9–15). Each measure was calculated as an average of the items with low score (1) and high score (4) representing low or high skill or attitude, respectively (Appendix 2).

The post-questionnaire included 20 items on a 1 to 4 Likert scale, based on two parts. The first two parts were identical to the pre-questionnaire with total of 15 items for instruction skills and attitudes assessments. The reliability of this part was 0.83, and the measures calculated were identical to the pre-questionnaire. The third part included five items on a 1 to 4 Likert scale related to the efficacy of the workshop. The reliability of this part was 0.81. An average of the items was calculated with low score (1) representing low efficacy and high score (4) indicating high efficacy (Appendix 2). Comparisons between pre- and post-measures were done using paired *t*-tests. Analysis of parallel items pre- and post-workshop was performed using Wilcoxon test for related samples.

The study was approved by institutional ethics committee (9687-12-SMC). All subjects signed consent form prior to participation in the workshop.

## Results

The sample included 48 subjects who replied to pre- and post-workshop questionnaires. Of them, data of 26 identified subjects were available on both time points. Data of 10 participants were available on the pre-only and an additional 12 on the post-only due to lack of participants’ awareness to complete both questionnaires (mostly at the first workshops) as well as because of technical obstacles during data collection. Response to the questionnaires was analyzed and a cutoff score of 85% was applied. One participant with 56% responses on the pre-questionnaire and 58% on the post-questionnaire was omitted. One additional participant completed 13 (87%) and 14 (93%) questions out of 15 on the pre-questionnaire. On the post–questionnaire, four participants completed 19 of 20 answers (95%). Thus, the analysis was based on 25 participants.

Background data indicated that 22 participants were residents (88%) and 3 were specialists. A variety of medical expertise was seen (highest were eight pediatricians and six internal medicine doctors). Their work experience ranged between 1 and 10 years. Twenty participants (80%) had previous instruction experience, and seven had previous experience as tutors.

**Fig. 4:**
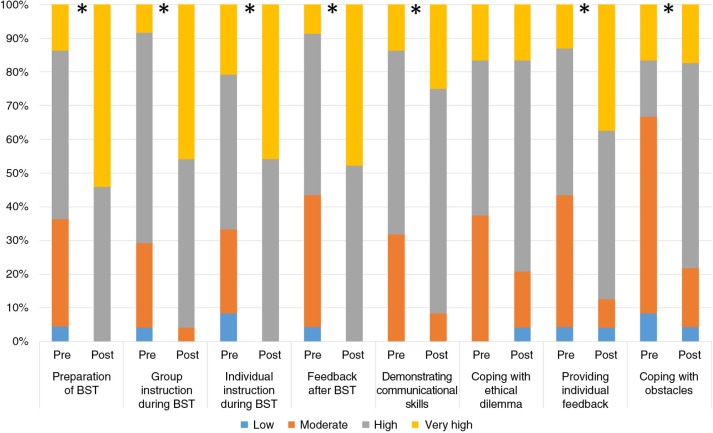
Teaching skills self-assessment: pre- versus post-workshop. Appendix 2, part 1; *N* = 25; *p < 0.05 Wilcoxon test.

Comparisons between pre- and post-measures were performed in two steps. First, mean scores of each questionnaire component (teaching skills and attitudes toward tutor's role) were compared using paired *t*-tests. Second, the score of each question was compared between pre- and post-questionnaires by Wilcoxon test. Significantly improved teaching skills were demonstrated upon workshop completion (mean 3.3, SD 0.5) compared with pre-training (mean 2.6, SD 0.6; *p<*0.001). Moreover, significant increase of self-evaluated skills was indicated in most examined parameters including preparation of BST, group and individual instruction during BST, feedback after BST, demonstrating communicational skills during patient–physician interactions as role model, providing individual feedback, and coping with student's problems (Appendix 2 and Fig. 4).

**Fig. 5:**
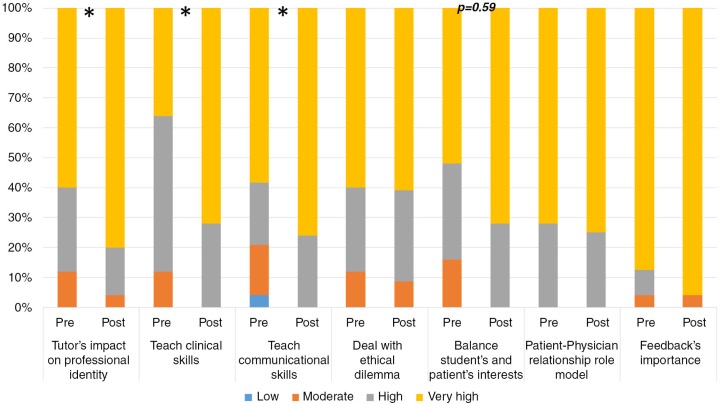
Practitioners’ perceptions regarding tutor's role during clinical education: pre- versus post-workshop. Appendix 2, part 2; *N* = 25; *p < 0.05 Wilcoxon test.

Assessment of participants’ attitudes revealed a general significantly improved internalization of tutor's roles after training completion (mean 3.7, SD 0.3) compared with pre-workshop (mean 3.5, SD 0.5; *p=*0.002). Additionally, significant increase was found regarding the tutor's central role during student professional identity development as well as to teach clinical and communicational skills concomitant with a tendency to significant increase regarding tutor's responsibility to balance between students’ benefit and patient's interest (Fig. 5).

Overall learning efficacy was scored as 3.7±0.5 on the questionnaire's third component indicating high efficacy. Specifically, BST preparations, BST, and student's feedback scores were 3.87±0.34, 3.74±0.45, and 3.74±0.6, respectively. Teaching communication skills and coping with ethical dilemma were assessed by the participants as 3.66±0.6 and 3.46±0.8, respectively.

Associations with background variables were performed as to instruction and previous experience as tutor, expertise level, and years of practice. No significant associations were indicated. Therefore, no adjusted analysis was done.

Reliability according to test–retest of skills or attitudes indicating significant associations of *r=*0.44 (*p=*0.027) for the skills and *r=*0.73 (*p<*0.001) for the attitudes measures. In the pre-questionnaire, no association between skills and attitudes was found, while in the post-questionnaire a significant association was indicated (*r=*0.42, *p=*0.036).

## Discussion

BST is a common format for medical teaching. Learners are taught in an interactive manner with real patients to acquire medical skills and interpersonal manners necessary for their daily practice as physicians (2). Educators today are required to have an expanded toolkit of teaching skills and clinical experience (16). The suggested model describes the stages of BST with continuing correlation between clinical teaching step and required communication tools used by medical teachers in order to coordinate effectively between patient and students. The model is flexible and can be easily adjusted to all levels of medical training such as students (during diverse stages of medical school) and interns.

The complexity of BST has been well recognized in the medical education literature (4). The situation in which a patient with medical illness is brought together with group of medical students under the scope of their professional and academic duties is extremely sensitive and sophisticated. The tutor, who stands in the middle of encounter, must be aware of the complexity and navigate with professionalism and humanism between patient's and students’ needs in order to achieve meeting's goals. Previous models for BST have been suggested in order to simplify BST. Cox established a stepwise model for BST starting from preparation to deriving work for the next time (9, 17–22). Janicik and Fletcher clarified certain domains with reference to barriers and advantages during BST (11). Our model has several similarities to these models such as its three-stage structure (preparation–BST–debriefing). On the other hand, the suggested model is unique since it deals simultaneously and consistently with both clinical and communication aspects throughout BST. Therefore, our model not only targeted on the *WHAT* and *WHEN* certain acts should be performed (e.g., individual followed by group teaching during BST) but also targeted on the *HOW* by defining clear communication tools for each step (e.g., leading questions, closed vs. open questions). Additionally, we developed original curriculum comprising of escalating scenarios and role model instruction within simulation room in order to support model implementation and optimize practitioners’ experience.

The combined clinical–communication approach during medical education is the fundamental principle (4) in our tutors training program. Although it is obvious that theoretical medical knowledge and clinical experience are mandatory for every physician, the importance of an open and safe patient–physician relationship is well established (2). Since the tutor stands in a central position as a teacher and a role model (14), we believe that tutors’ training should combine professionalism and humanism (4). Therefore, we emphasize that concept throughout the workshop beginning from the theoretical discussion and during BST model learning and implementation. We believe the stepwise approach during the consecutive scenarios with escalating clinical and communication objectives results in adequate model implementation as demonstrated by practitioners’ questionnaires.

The described results demonstrate a clear self-assessment improvement with regard to various teaching skills such as individual and group instruction during BST and feedback from students. In addition to skills mean score impressive increase from 2.6 to 3.3, a significant improvement was found in almost every assessed skill. Importantly, that improvement was not isolated to ‘pure’ clinical aspects of clinical teaching such as BST teaching, but also included communication parameters such as demonstrating communication skills during patient–physician interactions as a role model. These results may be seen as better internalization of various tutor's roles on both clinical and communicational aspects. Although most of the examined attitudes were highly scored prior to participation (mean 3.3 on the pre-questionnaire), significant increase was found in 3/7 examined parameters with additional trend (*p=*0.59) in an additional item.

The significant improvement at the post-questionnaires highlights the efficacy of the BST model during tutor training. Although we evaluated only practitioners’ self-assessment and not students’ opinion on their teaching skills as in a previous article (12), the impressive significant improvement demonstrated in most examined items supplies high reliability of our practitioners’ teaching skills and abilities to cope efficiently with the complexity of BST. Although self-assessment may not necessarily reflect external observations (23), it still remains a basic evaluation tool for medical education (24, 25).

In conclusion, successful BST involves the combination of advanced teaching and communication skills. During BST, the teacher serves as a coordinator and mediator between patient and students. Therefore, we see the medical teacher not only as an excellent clinician and motivated student instructor but also as a BST manager. The BST model is modular and suitable for diverse disciplines and clinical education situations in various learners’ levels so it can be implemented in all BST scenarios involving conscious patients. It can be taught and practiced with SP or patients, leading to better implementation during clinical rotation. We believe that adherence to the logical stepwise process will result with improved teaching skills in the challenging environment of BST, leading to highly professional and humanistic medical education.

## Appendix 1: Scenarios practiced during the workshop

**Template used during scenarios’ development:**

1. Simulation title
2. Duration
3. Participants
4. Objectives
5. Points for debriefing
6. Required achievements
7. Background: SP character, context, SP's emotional state and behavior
8. Clinical note
9. Scenario development with several optional directions according to participant's behavior
10. Decoration, required instruments

**Scenario number 1:**

That scenario had a single clinical objective without a communication challenge. The SP was a 61-year-old patient who had a cerebrovascular attack (CVA) 3 months prior to the current hospitalization that left him with hemiparesis. The ‘patient’ was re-hospitalized 2 days ago due to mild dysarthria that resolved spontaneously. The clinical objective was to teach the students the basic elements of neurological examination including level of consciousness, cranial nerves, as well as sensor and motor limbs assessment. The focus during that scenario was to implement the bedside teaching (BST) model step by step – single student practicing neurological examination followed by group teaching during which every student examined only the positive neurological signs (weakness of left limbs). The practicing student was instructed to perform a non-ordered neurological examination in order to let the practicing tutor instruct him in the appropriate way of examination. SP was instructed to be polite with positive attitude toward the tutors and students in order to avoid any additional communication challenge. That scenario lasted 10 min.

**Scenario number 2:**

That scene focused on a 63-year-old heavy smoker patient with previously diagnosed chronic obstructive pulmonary disease (COPD) who was hospitalized in the internal medicine department in order to conduct CT-guided biopsy of a coin lesion. That scenario included two clinical goals – to practice medical history taking as well as physical examination of the lungs. Therefore, the tutor was supposed to start personal instruction of a single student for history taking followed by group discussion on the topic. Thereafter, second student performed the physical examination followed by another group discussion according to the BST model. In addition to the double clinical objective, during that scenario we added a communication challenge – the patient was ‘notified’ 5 min prior to BST that his planned biopsy was postponed due to urgent cases. Therefore, the tutor had to cope with an angry patient at the beginning of the meeting. An additional communication increment was an emotional burden of the patient who had lost his father from lung cancer after smoking for decades. This scenario lasted 15 min.

**Scenario number 3:**

The third and last scenario was the most complicated. It included all three stages of the BST model and lasted 25 min. As opposed to the first two simulations, during this scenario workshop instructors did not join practitioners during the simulations in order to let them cope independently with SP. The simulation was recorded and debriefed after its completion by a video system specially designed for medical simulation at the Israel Center for Medical Simulation. The SP presented a 55-year-old patient who was admitted 2 days ago to the emergency room due to colonic obstruction acutely presented with severe abdominal pain and stool vomiting. He was transferred to the OR for laparotomy during which a colon cancer was diagnosed and treated by total colectomy with normal post-operative recovery. The clinical objective was to teach the concepts of post-operative evaluation and treatment. During physical examination, the SP demonstrated pain, which demanded an empathetic approach from the tutor while instructing the students. Additionally, the patient declared during BST that he refuses to be treated by chemotherapy; therefore, he presented an ethical dilemma to tutors and students. Moreover, an additional actor was added to the scenario as the practicing student who was instructed to confront the tutor during debriefing regarding the patient who refuses life-saving treatment. Therefore, this scenario had several challenges. First, it included a comprehensive implementation of the BST model step by step. Second, the tutor had to simultaneously cope with a patient suffering from severe disease after a major surgery (clinical challenge) and the ethical issue raised by the patient and later discussed with students (communication challenge).

## Appendix 2: Post-workshop Questionnaire^a^

**Table:**

Part 1: During the following questions, we will ask you to assess your ability. to implement the following teaching skills:	Low	Moderate	High	Very high
1. BST preparation – defining goals, patient selection, and students’ preparation	1	2	3	4
2. Group instruction during BST	1	2	3	4
3. Individual instruction during BST	1	2	3	4
4. Feedback after BST	1	2	3	4
5. Demonstrate communication skills during patient–physician interactions as a role model	1	2	3	4
6. Coping with ethical dilemma	1	2	3	4
7. Providing individual feedback	1	2	3	4
8. Coping with personal and disciplinary students’ difficulties	1	2	3	4

**Table:**

Part 2: Please comment on your position toward the following statements:	Not agree at all	Slightly agree	Moderately agree	Definitely agree
9. The tutor has a central role during student's professional identity development	1	2	3	4
10. The tutor's main role is to teach clinical skills	1	2	3	4
11. As a tutor my role is to teach interpersonal communication skills as a part of medical education	1	2	3	4
12. It is important to deal with ethical dilemmas from early stages of clinical medical exposure	1	2	3	4
13. The tutor is responsible for the balance between students’ benefit and patient's interest	1	2	3	4
14. My attitude toward a patient may serve as an example for patient–physician relationship	1	2	3	4
15. Individual teaching and feedback are important tools during clinical teaching	1	2	3	4

**Table:**

Part 3:The following questions assess the extent to which the workshop assisted you to improve the following skills:	Not at all	Slightly	Moderately	Very much
16. BST preparation	1	2	3	4
17. BST	1	2	3	4
18. Feedback to students	1	2	3	4
19. Teaching communication skills	1	2	3	4
20. Coping with ethical dilemmas	1	2	3	4

BST, bedside teaching.

a The first two components (questions 1–8 and 9–15) were identical to the pre-workshop questionnaire. The last component (questions 16–20) was administered only after workshop's completion.
